# Low atrial septal pacing with a handmade stylet when right atrial appendage pacing is difficult: A case series

**DOI:** 10.1016/j.hrcr.2024.02.014

**Published:** 2024-02-28

**Authors:** Daisuke Yamazaki, Takahide Fujihashi, Hirokazu Amamizu, Toru Takahashi

**Affiliations:** Department of Cardiology, Akita Cerebrospinal and Cardiovascular Center, Akita, Japan

**Keywords:** Pacemaker implantation, Low atrial septal pacing, Right atrial appendage pacing, Conventional stylet, Handmade stylet

**Key Teaching Points**

• Even when the amplitude and threshold of the P wave is poor and atrial lead placement in the right atrial appendage is difficult, the low atrial septum may have good wave amplitude and threshold.
• If low atrial septal pacing can be performed with a conventional stylet, the pacing site can be easily changed.
• The trick is to bend the stylet in a large arc so when the atrial lead is withdrawn from the inferior vena cava into the right atrium, the lead tip contacts the atrial wall and does not enter the right ventricle.

## Introduction

Dual-chamber pacemakers are implanted for symptomatic sick sinus syndrome and atrioventricular block. In many cases, atrial leads are implanted in the right atrial appendage (RAA) during dual-chamber pacemaker implantation because of the simplicity of the procedure. However, there may be no lead placement site with good wave amplitude and threshold owing to fibrosis and remodeling of the RAA. In such cases, the options are high atrial septal pacing with pacing near the Bachmann bundle or low atrial septal (LAS) pacing with pacing near Koch’s triangle, but fewer delivery catheters are suitable for the atrial septum than the ventricular septum. Locators (Abbott, Chicago, IL) are also not often readily available. We report 12 cases in which atrial leads were placed in the LAS using a conventional stylet because there was no site for placement of an atrial lead owing to poor RAA parameters.

## Case report

When implanting a dual-chamber pacemaker at our institution, the RAA is the first choice of site for atrial lead placement. However, when the wave amplitude in the RAA is less than 1.0 mV or the threshold is greater than 1.0 V (pulse width 0.4 ms), the atrial lead is placed in the LAS using a conventional stylet. The pacemaker implantation procedure is as follows. Venography is performed from the left upper extremity and a pacemaker pocket is created in the left side of the chest. After puncturing the left axillary vein, 2 6–7F sheaths are inserted and a ventricular lead (active retractable fixation lead) is screwed in the ventricular septum using a handmade stylet. We then attempt to place an atrial lead (active retractable fixation lead) in the right auricle. If the wave amplitude in the RAA is less than 1.0 mV or the threshold is greater than 1.0 V (pulse width 0.4 ms), the atrial lead implantation site is changed to LAS. The method of LAS lead placement is as follows.

**Figure 1:**
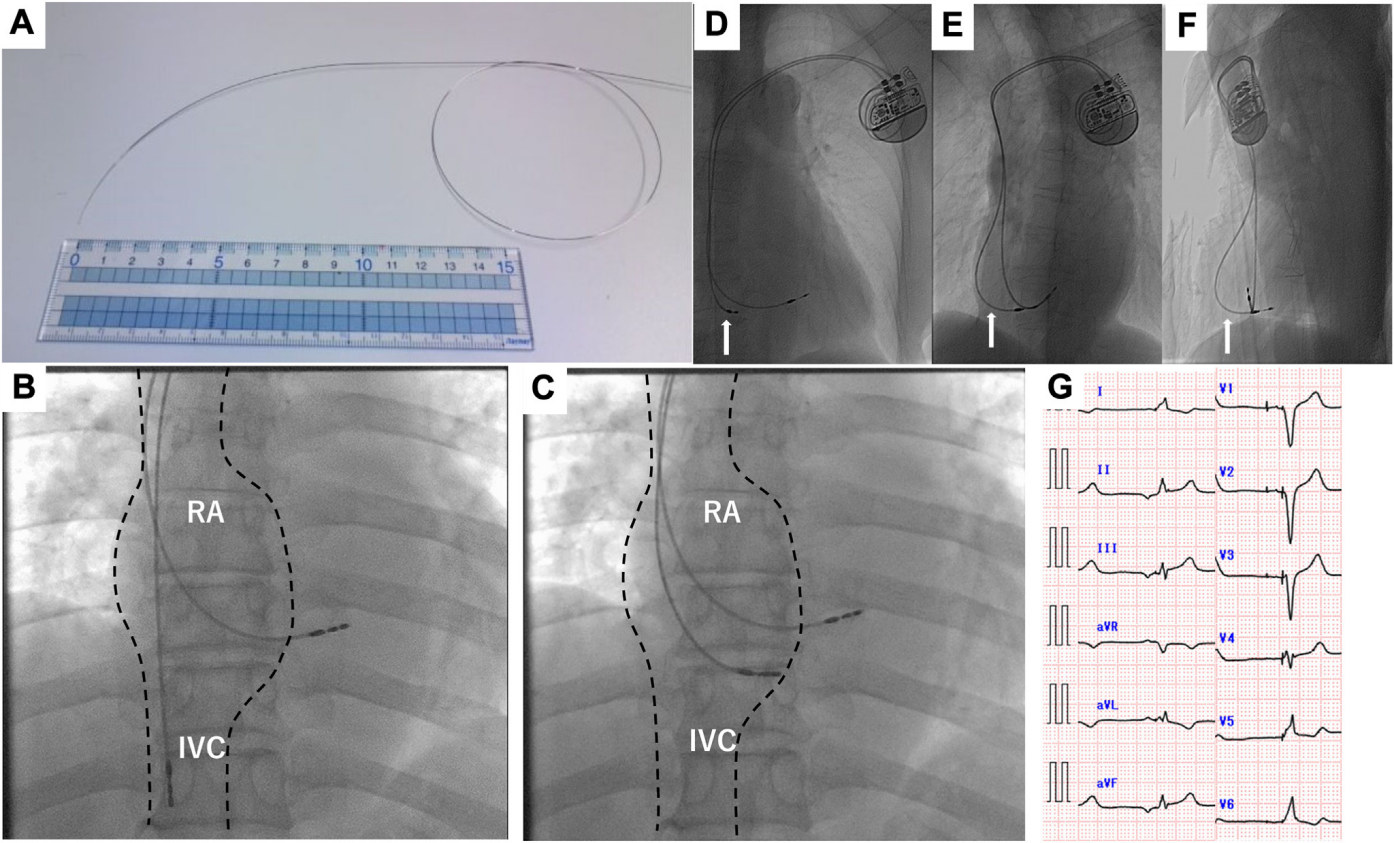
Method of stylet bending by hand for low atrial septal (LAS) implantation. **A:** A curve is created in a large arc so the lead tip contacts the right atrial wall (but does not enter the right ventricle) when the atrial lead is withdrawn from the inferior vena cava. It is important not to overbend the stylet tip. **B:** The atrial lead is advanced into the inferior vena cava and the handmade stylet is inserted and then withdrawn while applying counter-clockwise rotation (posterior-anterior view). **C:** When the atrial lead tip enters the right atrium, it contacts the wall near Koch’s triangle. When the lead is pressed slightly, the lead tip moves up and down according to the contraction of the right atrium (posterior-anterior view). **D:** After pacemaker implantation. The arrow indicates the atrial lead (posterior-anterior view). **E:** After pacemaker implantation. The arrow indicates the atrial lead (left anterior oblique view). **F:** After pacemaker implantation. The arrow indicates the atrial lead (lateral view). **G:** Electrocardiogram of LAS pacing. P waves are negative in II, III, aVF and positive in V_1_ induction. RA = right atrium; IVC = inferior vena cava.

After the atrial lead is dropped into the inferior vena cava (IVC), the stylet is bent gently enough to prevent the atrial lead from entering the right ventricle (RV), as shown in Figure 1A. The stylet is inserted and the lead is pulled from the IVC into the right atrium (RA) while applying counter-clockwise torque, so the tip of the lead contacts the LAS site just above the coronary sinus ostium (near Koch’s triangle) (Supplemental Video). The wave amplitude and threshold are measured at that site and if they are within acceptable range, the lead is screwed in and fixed. If the data are not acceptable, the lead is returned to the IVC. The lead is manipulated in the same way with the 2 cm stylet tip pointing slightly backwards, and this is repeated until a site with acceptable parameters is found. Implantation of the atrial lead in the LAS is confirmed by fluoroscopy and electrocardiogram.^1^

The fluoroscopic criteria for LAS pacing are as follows: the atrial lead tip is facing the spine in the left anterior oblique position and the atrial lead is bent at 90°, as shown in Figure 1E.

The electrocardiogram criteria for LAS pacing are as follows: P waves are negative in II, III, and aVF and positive in V_1_ induction, as shown in Figure 1G.

All data were statistically analyzed using EZR software, which is based on R and R Commander. Continuous variables are presented as the mean ± standard deviation if normally distributed according to a test of normality (Kolmogorov–Smirnov test) or as the median (interquartile range) if not normally distributed.

## Results

Twelve patients underwent LAS pacing between March 2021 and July 2023. LAS pacing was performed in 23.5% of all patients undergoing pacemaker implantation.

Table 1 lists the patients’ baseline characteristics and procedural outcomes. Mean age was 83 (78.5–84.3) years, with many older patients. The following pacemaker leads were used: 5076 CapSureFix Novus MRI (Medtronic, Minneapolis, MN), 7840 INGEVITY plus (Boston Scientific, Marlborough, MA), Solia S (Biotronik, Berlin, Germany), and Tendril STS (Abbott). LAS pacing was successful in all patients. Pacemaker implantation procedure time (from local anesthesia to completion of closure) was 110.1 ± 31.5 minutes. There were no complications related to the procedure, such as pacemaker infection, dislodgement, or hematoma. At the time of pacemaker implantation, atrial lead parameters were stable with wave amplitude of 2.5 ± 1.4 mV, threshold of 0.76 (0.4–0.81) V (pulse width 0.4 ms), and impedance of 570 ± 153 Ω.

**Table 1:** Patient backgrounds and procedure outcomes

Case no.	Age	Sex	Arrhythmias	LAD (mm)	Success	Atrial lead	Procedure	Fluoroscopy	Fluoroscopy	ECG criteria	P-wave ECG criteria	Amplitude	Threhold	Impedance
							Time (min)	Time (min)	criteria	Ⅱ, Ⅲ, aVF	V_1_	(mV)	(V)	(Ω)
1	83	Male	SSS	49.3	Yes	CapSureFix Novus MRI 52 cm	160	38.6	Yes	Negative	Flat	0.5	1.6	588
2	83	Male	SSS	47	Yes	INGEVITY plus 45 cm	170	37	Yes	Negative	Positive	5	0.4	600
3	77	Male	SSS	45.9	Yes	Solia S 45 cm	100	13.7	Yes	Negative	Positive	0.7	1	561
4	80	Female	SSS	31.8	Yes	Tendril STS 46 cm	140	32.7	Yes	Negative	Positive	3.1	1.5	438
5	88	Male	SSS	32.5	Yes	CapSureFix Novus MRI 52 cm	120	35.1	Yes	Negative	Flat	2.4	0.5	437
6	48	Male	SSS	32.8	Yes	CapSureFix Novus MRI 45 cm	81	12.9	Yes	Negative	Positive	2.8	0.4	551
7	86	Female	AV block	33.3	Yes	Tendril STS 46 cm	84	13.4	Yes	Negative	Positive	2	0.75	390
8	83	Female	AV block	36.3	Yes	Tendril STS 46 cm	78	10.8	Yes	Negative	Flat	5	0.75	440
9	85	Female	SSS, AV block	43.4	Yes	CapSureFix Novus MRI 45 cm	84	13.3	Yes	Negative	Positive	1.25	0.7	475
10	73	Female	AV block	28.8	Yes	INGEVITY plus 45 cm	89	15.3	Yes	Negative	Positive	2.5	0.4	700
11	84	Male	AV block	35.3	Yes	INGEVITY plus 45 cm	103	24.7	Yes	Negative	Flat	2	0.7	900
12	79	Male	AV block	39	Yes	INGEVITY plus 45 cm	113	15	Yes	Negative	Positive	3.3	0.4	763

AV = atrioventricular; ECG = electrocardiogram; LAD = left atrial diameter; SSS = sick sinus syndrome.

Case 1 had a P-wave amplitude of less than 1.0 mV and a threshold of more than 1.0 V, and procedure time was long (160 minutes) owing to the time required to determine the atrial lead placement site. Case 4 also had a long procedure (140 minutes) because no pacing site could be found below the threshold of 1.0 V.

**Figure 2:**
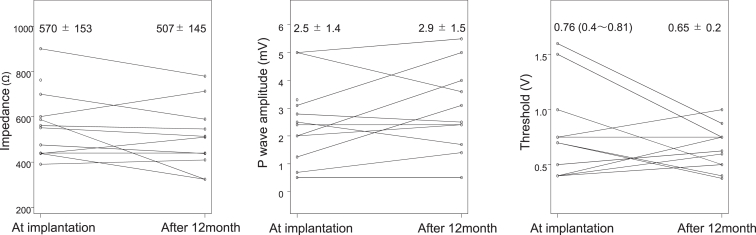
Changes in atrial lead parameters between the time of implantation and after 12 months.

The time course of atrial lead parameters for LAS pacing is shown in Figure 2. The parameters at 12 months were stable, with resistance of 507 ± 145 Ω, P-wave amplitude of 2.9 ± 1.5 V, and threshold of 0.65 ± 0.2 V (pulse width 0.4 ms).

## Discussion

This case series demonstrates that in patients in whom it is difficult to place the atrial lead into the RAA, an atrial lead can be placed into the LAS using a handmade stylet, with good subsequent progress in lead parameters. The lack of a suitable site for pacing parameters in the RAA can occur in routine practice; however, to the best of our knowledge, there are no previous reports of changing the atrial pacing site in such cases. In other words, reports on atrial septal pacing have all described targeting the atrial septum from the outset.

Compared to apex pacing, ventricular septal pacing is reported to be less prone to myocardial remodeling and induction of heart failure, for the reason of less asynchrony between the left ventricle and both ventricles.^2^^,^^3^ Therefore, ventricular septal pacing is widely used.

In LAS pacing, the pacing site is close to the atrioventricular node, which shortens the PQ time during atrial pacing and facilitates avoidance of ventricular pacing.^4^ Atrial septal pacing improves synchrony of contraction in both atria.^5^ For this reason it has been reported to reduce the occurrence of atrial fibrillation^6^; however, atrial septal pacing is performed less frequently than ventricular septal pacing because it has not been reported to be as effective in reducing heart failure as ventricular septal pacing and because RAA pacing is an easier procedure.

Other than the RAA, it is easy to place an atrial lead in the lateral RA. However, this site is not recommended because of the risk of atrial wall perforation and contralateral pneumothorax owing to the inherent thinness of the atrial wall (1–2 mm) and as the helix of an active retractable fixation lead is about 2.0 mm long.^7^ There are 2 methods for LAS pacing, using the Locator (Abbott)^8^ or the Select Secure System (Medtronic).^9^ However, the Locator cannot be used immediately when a pacemaker from another manufacturer is implanted. In addition, when an atrial lead is placed in the RAA, an active retractable fixation lead is used, so a new 4.1F lumenless active-fixation lead is required when using the Select Secure System, which is not practical. LAS pacing can be attempted using a delivery catheter for the ventricular septum that is compatible with an active retractable fixation lead, but there is also a risk of bleeding after sheath removal, as it has to be replaced with a thick outer sheath. There are 3 anatomical sites of transseptal conduction from the RA to the left atrium: the Bachmann bundle, the fossa ovalis, and near the coronary sinus.^10^ Even when the RAA is remodeled and fibrotic and atrial lead placement is difficult, the atrial septum may have good wave amplitude and threshold, as in the present case series. High atrial septal pacing is a difficult technique because the lead must be fixed toward the ceiling of the RA. In contrast, LAS pacing is a simpler technique, as the lead can be pushed during lead fixation. A well-known problem that may be encountered during LAS pacing procedures or in follow-up is far-field sensing caused by the proximity of the atrial leads to the RV. The manufacturer’s normal settings are often fine, but if far-field sensing needs to be avoided, the atrial sensitivity setting can be blunted or the postventricular atrial blanking can be lengthened. In 12 cases in this case series, right ventricular waves were present in the intracardiac potentials of the atrial leads, but did not result in atrial oversensing with normal postventricular atrial blanking settings and atrial sensitivity settings. This method of LAS pacing with a handmade stylet allows the atrial implantation site to be changed easily with only a conventional stylet, without the need for an additional device. To place a lead in the LAS using this method, (1) the tip of the atrial lead must strike the atrial wall when the lead is pulled up and enter the RA through the IVC, and (2) the tip of the lead must not enter the RV when the lead is pulled up into the RA. The knack is therefore to bend the stylet in such a way that the lead tip points downwards while drawing a large arc, as shown in Figure 1A. When pulling the lead up, counter-clockwise rotation is applied so the lead tip points toward the atrial septum (behind). If the wave amplitude or threshold is poor, the tip should be bent back slightly (by about 2 cm) to change the point of contact slightly.

Das and colleagues^11^ also reported on LAS pacing with a handmade stylet in 2017. In this literature, the atrial lead is advanced into the RA and then under left anterior oblique view to target the LAS. The size of the RA and the height of the coronary sinus vary from case to case, so the bending of the stylet and the technique require familiarity. Our method is to aim at the LAS by pulling up the atrial lead from the IVC wall to the RA wall in a continuous tracing fashion. The trick is to make a gentle arc in the proximal stylet for continuous tracing and not to bend the tip too much so that the tip of the lead does not enter the RV and the tip of the lead points downward. The difference in this method is reflected in the difference in the curve of the stylet.

Continuation of the policy of LAS pacing for patients who have difficulty with RAA pacing will allow long-term outcome and parameter data to be accumulated, which may demonstrate not only the simplicity of the procedure but also the effectiveness of postoperative management. As patients undergoing pacemaker implantation become older, and the number of cases with poor wave amplitude and threshold in RAA may increase in the future, the ability to perform LAS pacing with a conventional stylet will become increasingly necessary.

## Conclusion

LAS pacing with a handmade stylet can be performed easily without the use of new devices, and the atrial lead parameters are stable after implantation.

## Disclosures

The authors have no conflicts of interest to disclose.

## Appendix Supplementary Data

Supplemental Video An atrial lead with a handmade stylet inserted is withdrawn from the inferior vena cava, while applying counter-clockwise rotation. The tip of the atrial lead enters the right atrium and contacts the wall near Koch's triangle. A slight push on the lead causes the lead tip to move up and down in time with the contraction of the right atrium (posterior-anterior view).
